# Rifampin-Releasing Triple-Layer Cross-Linked Fresh Water Fish Collagen Sponges as Wound Dressings

**DOI:** 10.1155/2020/3841861

**Published:** 2020-10-16

**Authors:** Jan Miroslav Hartinger, Peter Lukáč, Mikuláš Mlček, Michaela Popková, Tomáš Suchý, Monika Šupová, Hynek Chlup, Lukáš Horný, Jan Závora, Václava Adámková, Ondřej Slanař, Petr Kozlík, Katarina Molnarova, Eva Honsová, Lukáš Lambert, Tomáš Grus

**Affiliations:** ^1^Department of Clinical Pharmacology and Pharmacy, Institute of Pharmacology, First Faculty of Medicine, Charles University and General University Hospital in Prague, Prague, Czech Republic; ^2^2nd Department of Cardiovascular Surgery, First Faculty of Medicine, Charles University and General University Hospital in Prague, Prague, Czech Republic; ^3^Institute of Physiology, First Faculty of Medicine, Charles University and General University Hospital in Prague, Prague, Czech Republic; ^4^Department of Composites and Carbon Materials, Institute of Rock Structure and Mechanics, Academy of Sciences of the Czech Republic, Prague, Czech Republic; ^5^Department of Mechanics, Biomechanics and Mechatronics, Faculty of Mechanical Engineering, Czech Technical University in Prague, Prague, Czech Republic; ^6^Institute of Medical Biochemistry and Laboratory Diagnostics, First Faculty of Medicine, Charles University and General University Hospital in Prague, Prague, Czech Republic; ^7^Department of Analytical Chemistry, Faculty of Science, Charles University, Prague, Czech Republic; ^8^Clinical and Transplant Pathology Centre, Institute of Clinical and Experimental Medicine, Prague, Czech Republic; ^9^Department of Radiology, First Faculty of Medicine, Charles University and General University Hospital in Prague, Prague, Czech Republic

## Abstract

**Objectives:**

Surgical wounds resulting from biofilm-producing microorganisms represent a major healthcare problem that requires new and innovative treatment methods. Rifampin is one of a small number of antibiotics that is able to penetrate such biofilms, and its local administration has the potential to serve as an ideal surgical site infection protection and/or treatment agent. This paper presents two types (homogeneous and sandwich structured) of rifampin-releasing carbodiimide-cross-linked fresh water fish collagen wound dressings.

**Methods:**

The dressings were prepared by means of the double-lyophilization method and sterilized via gamma irradiation so as to allow for testing in a form that is able to serve for direct clinical use. The mechanical properties were studied via the uniaxial tensile testing method. The *in vivo* rifampin-release properties were tested by means of a series of incubations in phosphate-buffered saline. The microbiological activity was tested against methicillin-resistant *staphylococcus aureus* (MRSA) employing disc diffusion tests, and the *in vivo* pharmacokinetics was tested using a rat model. A histological examination was conducted for the study of the biocompatibility of the dressings.

**Results:**

The sandwich-structured dressing demonstrated better mechanical properties due to its exhibiting ability to bear a higher load than the homogeneous sponges, a property that was further improved via the addition of rifampin. The sponges retarded the release of rifampin *in vitro*, which translated into at least 22 hours of rifampin release in the rat model. This was significantly longer than was achieved via the administration of a subcutaneous rifampin solution. Microbiological activity was proven by the results of the disc diffusion tests. Both sponges exhibited excellent biocompatibility as the cells penetrated into the scaffold, and virtually no signs of local irritation were observed.

**Conclusions:**

We present a novel rifampin-releasing sandwich-structured fresh water fish collagen wound dressing that has the potential to serve as an ideal surgical site infection protection and/or treatment agent.

## 1. Introduction

Surgical wound infections constitute one of the most frequently occurring postsurgical complications and often lead to prolonged hospital stays, increased healthcare costs, and a deterioration in the overall treatment results [1]. Perioperative local antibiotic treatment has been widely clinically studied to date with generally promising results [2–5]. While most such studies have considered gentamicin [2] or vancomycin [3, 4], it has been suggested that the administration of systemic rifampin improves the outcome in deep sternal wound infections caused by staphylococci following cardiac surgery [6] as well that of staphylococcal infections associated with stable orthopedic implants [7]. The local application of 250 mg of rifampin over the mesh during the inguinal hernia repair procedure has been reported to significantly reduce the occurrence of postoperative surgical site infection [5] without the need for systemic administration. Rifampin penetrates readily through the biofilm and is often recommended for the treatment of surgical site infections together with other antibiotics that act to prevent the rapid development of rifampin resistance [8–10]. Since around 80% of surgical site infections are associated with the formation of a poorly permeable biofilm [8], a rifampin-releasing local wound dressing would serve as an ideal protection against, and for the treatment of, surgical site infections.

Since antibiotic-releasing collagen-based wound dressings have been proven to be effective in terms of the prevention and treatment of postoperative wound infection [11], we hypothesized that the combination of a collagen-based wound dressing with rifampin would also be beneficial in the case of surgical wound treatment. Various types of collagen scaffolds/sponges have been described in the literature as suitable wound dressings [12] that can be employed as carriers of antibiotics [11]. Grzybowski et al. created a two-layered dressing with membrane and sponge layers. They applied aminoglycosides between these who layers so as to produce a sponge which allowed for the controlled release of the antibiotics. They also proved that the dressing was effective in terms of the reduction of a *pseudomonas aeruginosa* colony-forming unit (CFU) in a mouse model of a wound infection [13].

To the best of our knowledge, no collagen sponges containing rifampin have been used regularly to date in the clinical setting. Parker et al. conducted an experiment with a rifampin-soaked fibrinogen-implanted collagen sponge and suggested that the rifampin is partially bonded to the sponge after the sponge had been immersed in a rifampin solution. Only 75% of the rifampin was washed out during elution testing. They also measured rifampin concentrations in the blood of rats that had been treated with the sponges and determined that the concentration remained higher than 0.001 mg/L for 6 hours [14].

Most studies conducted on collagen dressings have considered collagen of mammal origin which induces an immune reaction in 3-4% of the treated population [15]. It has been proven that the use of fresh water fish scale-derived collagen is advantageous due to its enhanced biocompatibility and high level of direct cell adhesion [16]. Since fish collagen loses stability at body temperature [17], it has to be cross-linked in order to maintain its spongy consistency when used as a collagen dressing [16, 18]; carbodiimide has been demonstrated to provide a highly biocompatible collagen cross-linking agent [16, 19, 20].

We previously tested a homogeneous spongy matrix based on gentamicin- and vancomycin-containing homogeneous fresh water fish collagen cross-linked with carbodiimide as a wound dressing in a rat surgical site infection model [21, 22]. The presented study also involved the further modification of the structure of the sponge and the testing of its characteristics as a potential carrier of rifampin. A homogeneous collagen sponge and a three-layered sandwich-structure sponge were tested *in vivo* and *in vitro* with respect to their mechanical properties, stability in aqueous environments, and their *in vitro* and *in vivo* rifampin release properties. In addition, the histological reaction to the collagen sponges was investigated employing a rat model.

## 2. Materials and Methods

### 2.1. Preparation of Homogeneous and Sandwich Collagen Sponges

**Figure 1:**
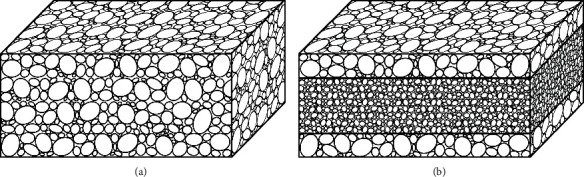
Simplified scheme of (a) highly porous homogeneous sponge and (b) sandwich sponge with rigid core with lower porosity and highly porous peripheral layers.

Homogeneous sponges and sandwich sponges (Figure 1) were prepared from collagen type I isolated from the skin of fresh water fish (*cyprinus carpio*, Třeboň carp, Třeboň fishery, Czech Republic, controlled breeding). The isolation and extraction of the collagen have been described previously by Suchý et al. [23]. The homogeneous sponges were prepared from an aqueous collagen dispersion (1 wt%) acquired via the swelling of the collagen in deionized water (10°C, 3 hours), which was homogenized using a disintegrator (10,000 rpm, 10 min), left for 20 minutes at room temperature, and homogenized for a second time (10,000 rpm, 5 min). The resulting dispersion was placed in containers, frozen at a temperature of -80°C for 6 hours, and then lyophilized (BenchTop 4KZL, VirTis, USA). The sandwich sponges were prepared via the following procedure: an aqueous collagen dispersion was prepared by means of the swelling of the collagen in deionized water (10°C, 3 hours), which was homogenized using a disintegrator (10,000 rpm, 10 min), left for 20 minutes at room temperature, and homogenized for a second time (10,000 rpm, 5 min). The collagen dispersion concentration of the core was 5 wt% while that of the peripheral layers was 1 wt%. The resulting 5 wt% dispersion was placed in containers, frozen at a temperature of -80°C for 6 hours, and then lyophilized, and subsequently, the core was impregnated with the resulting 1 wt% dispersion in separate containers and left for 3 hours at room temperature, frozen at a temperature of -80°C for 6 hours, and then lyophilized.

The stability of the homogeneous and sandwich collagen sponges was enhanced via cross-linking with a 95 wt% ethanol solution containing EDC (N-(3-dimethylaminopropyl)-N-ethylcarbodiimide hydrochloride) and NHS (N-hydroxysuccinimide) at a molar ratio of 4 : 1. The EDC and NHS were used as received (Sigma-Aldrich, Germany). 0.625 g of the EDC was applied for 1 g of collagen and mixed with 150 mL of 95 wt% ethanol solution (Penta, Czech Republic). Following a reaction period of 3 hours at 37°C, the sponges were washed in 0.1 M Na_2_HPO_4_ (for at least 30 min), followed by rinsing with deionized water (for at least 30 min), frozen at -30°C for 6 hours, and lyophilized.

Finally, the cross-linked homogeneous and sandwich collagen sponges were impregnated with a 95 wt% ethanol solution containing a weighed amount of rifampin natrium (Eremfat, RIEMSER Pharma GmbH, Germany) so as to attain a final weight ratio of the collagen to the pure rifampin of 5 : 4. The impregnated sponge was frozen at -80°C and lyophilized again. The final stage involved the cutting of samples of the appropriate dimensions. The final amount of rifampin was verified following the conclusion of the preparation phase (see In Vitro Pharmacokinetic Tests), i.e., prior to the subsequent testing phase.

### 2.2. Scanning Electron Microscopy

Scanning electron microscopy (SEM) was conducted in the high vacuum mode using a Quanta 450 electron microscope (FEI, USA). The samples were coated with a thin layer of gold in an ion sputter (Emitech K550X, Quorum Technologies, U.K.).

### 2.3. Testing of the Mechanical Properties of the Collagen Sponges

**Figure 2:**
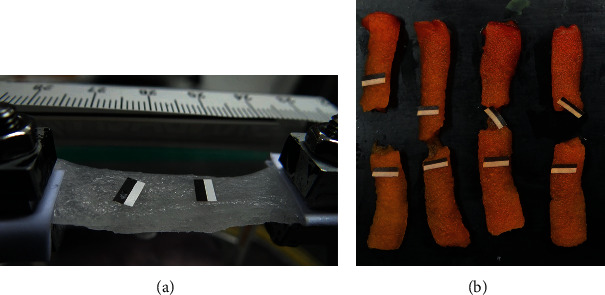
Samples of the homogeneous collagen sponges without rifampin (a) and with rifampin (b).

**Figure 3:**
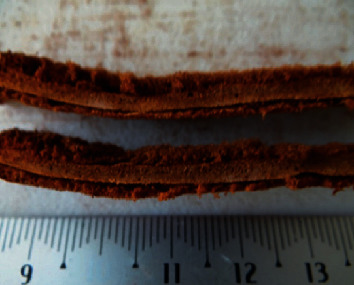
Samples of the sandwich-structured collagen sponges with rifampin.

**Figure 4:**
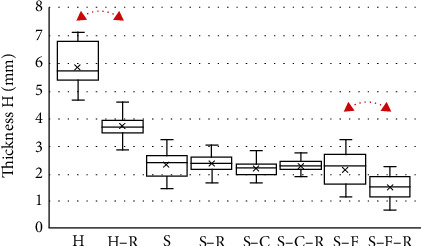
Thicknesses of the tested collagen sponges (*N* = 10): homogeneous collagen sponges (H), sandwich-structured sponges (S), sandwich-structured sponges—core material (S-C), sandwich-structured sponges—face sheet (S-F), sponges with rifampin (-R). The arrows denote statistically significant differences between identical sponges with or without rifampin (*t*-test, *p* < 0.05).

The mechanical properties of the collagen sponges were studied by means of uniaxial tensile tests. The experiments are aimed at elucidating the effect of the addition of rifampin and the design of the sponge on the mechanical integrity. Experiments were performed on homogeneous collagen sponges (H) (Figure 2(a)), homogeneous collagen sponges with rifampin (H-R) (Figure 2(b)), sandwich-structured collagen sponges without (S) and with rifampin (S-R) (Figure 3), the core material of the sandwich structure without (S-C) and with rifampin (S-C-R) and, finally, with the face sheet material of the sandwich sponges, again without (S-F) and with incorporated rifampin (S-F-R). The sponges took the form of lyophilized layers from which rectangular strips were cut. The samples were hydrated in phosphate-buffered saline (PBS, pH ~7.4, Sigma Aldrich, St. Louis, Missouri, USA) prior to the mechanical testing; Figure 2 depicts the typical geometry of the samples. The dimensions were determined via the image analysis of photographs taken before the commencement of the experiments. Each of the dimensions was determined at five different positions on the sample. Figure 3 illustrates the natural variability of the thickness of the sponges, a summary of which is provided in Figure 4.

Tensile testing was conducted using a Zwick/Roell multipurpose testing machine with HBM U9C 50 N load cell which was connected in ±25 N regime (tension/compression). HBM U9C is in 0.2 accuracy class. The testing machine was also equipped with a built-in videoextensometer. The stress-strain relationships were obtained from the force-displacement data recorded on a PC during loading. The nominal stress, *σ*, was calculated as the ratio of the current force to the reference cross-sectional area determined prior to experimentation. The deformation, *ε*, was expressed as the ratio of the displacement, measured using the videoextensometer, to the distance between marks that were created on the surface of the samples prior to experimentation (see Figure 2).

Since the mechanical behavior of collagenous material in the hydrated state is known to be nonlinear, the comparison of the mechanical properties of the sponges based on the Young's modulus determined at *ε* = 0.01and *E*_0.01_ and at *ε* = 0.1and *E*_0.1_. *E*_0.01_ was considered to be the modulus of elasticity corresponding to small deformation regions, whereas *E*_0.1_ characterized the stiffness of the material in the large strain regions; both were determined as the slope of the tangent made to the *σ*–*ε* curve. Ten samples were mechanically tested for each group of samples.

### 2.4. Rifampin Stability Tests

Since the stability of rifampin at 37°C is limited [24], we performed the stability testing of rifampin in PBS at 37°C protected from light. We incubated solutions with 3 differing rifampin concentrations (600 mg/L, 60 mg/L, and 6 mg/L) in PBS under such conditions. The samples were drawn after 0 and 4 hours and after 1, 2, 6, and 7 days of incubation and subsequently analyzed using HPLC for the determination of the rifampin concentration.

### 2.5. Rifampin Release Tests

The release test was performed separately for the homogeneous and sandwich sponges. Sections of the collagen sponges with dimensions of 10 × 20 mm were studied. Six sections were obtained from three different sponges (three from the edges and three from the central parts) in order to test the variability of the rifampin content between the sponges and the homogeneity of the rifampin content within the sponges. The weight of the tested samples was 33.7 ± 7.4 mg for the homogeneous sponges and 43.3 ± 2.7 mg for the sandwich sponges (mean ± SD). Each of the tested sections was submerged in 20 mL of 37°C PBS in a test tube and gently rocked in an incubator at 37°C. The sponge sections were transferred to a newly tempered 20 mL of PBS after 15, 30, 60, 120, 180, 240, and 360 minutes, 24 hours, and 8 days of incubation. Samples of PBS with the released rifampin were subsequently analyzed using HPLC for the determination of the rifampin concentration. We discovered that the amount of the eluted rifampin was proportional to the weight rather than to the size of the samples (see Results). This information was used for dosing in the *in vivo* experiments.

### 2.6. Disc Diffusion Tests

The tests were performed separately for the homogeneous and sandwich sponges. Nine discs (6 mm in diameter) were cut from the sponges. The microbiological response was tested via the standard disc diffusion test with methicillin-resistant *staphylococcus aureus*, strain CNCTC 6271 (MRSA).

### 2.7. Animals and Catheter Implantation

With respect to the *in vivo* pharmacokinetic tests, we used adult male Wistar rats with median weights of 390 g (IQR = 362 − 411 g), 375 g (IQR = 353 − 390 g), and 379 g (IQR = 360 − 397 g) for the sandwich sponge group (S, *N* = 6), homogeneous sponge group (H, *N* = 6), and control group with subcutaneous (s. c.) rifampin (C, *N* = 8), respectively. The differences in the weights between the groups were not statistically significant. Intra-arterial catheters were implanted into the carotid arteries for the purpose of repeated blood sampling according to our standard protocol described in detail elsewhere [25]. Briefly, the catheters were implanted after 6-8 hours of fasting in aseptic conditions. The anesthesia took the form of the administration of 10 mg/kg of xylazin and 80 mg/kg of ketamin intramuscularly; no perioperative antibiotic prophylaxis was used in the study. The rats were placed in separate cages following catheter implantation with free access to food and water.

The food consisted of 90% of maintenance solid mixture diet for laboratory rats (Altromin International, Im Seelenkamp 20, D-32791 Lage, Germany) and 10% of powdered charcoal (Carbosorb, IMUNA PHARM, Jarková 17, 082 22, Šarišské Michaľany, Slovakia) in order to disrupt the enterohepatic circulation of the rifampin. 5% of charcoal added to standard food was previously proven to lower bilirubin levels in rats due to the adsorption of bilirubin in the gut and the disruption of its enterohepatic recirculation [26]. The rats were kept in separate cages until the conclusion of the experiment so as to prevent damage to the cannulas. The cannulas were heparinized following each sampling campaign. The rats were administered nadroparine once daily so as to prevent the formation of thrombi in the catheters. The rats were anesthetized following the final sample draw by means of isoflurane and euthanized using a combination of embutramide, mebezonium, and tetracaine (T-61, Intervet International B.V., Wim de Körverstraat 35, 5831 AN Boxmeer, Netherlands). Samples intended for the histological examination were excised from the administration site following euthanasia.

In order to exclude the influence of tissue damage during the sham surgery incision and subsequent administration of rifampin on the histological examination, an additional control (C2) group of 6 female Wistar rats was formed for the study of the effect of rifampin on local tissues. The rats were administered a rifampin solution subcutaneously at the same dose as for the other groups with no incision. Tissue from the administration site was used for the histological examination.

The experimental protocol was approved by the Institutional Animal Expert Committee of the First Faculty of Medicine, Charles University, and was performed at the University's accredited laboratory in accordance with Czech Act No. 246/1992 Coll. on the protection of animals against cruelty. All the animals were treated and cared for in accordance with European Guidelines on Laboratory Animal Care.

### 2.8. In Vivo Pharmacokinetic Tests

The pharmacokinetic study was initiated 72 hours following catheter implantation on those rats that had fully recovered from the catheter implantation procedure. An approximately 1 cm long incision was made on the backs of the rats. With respect to the S and H groups, a piece of rifampin-containing collagen sponge was inserted into the pocket and the wound was closed with a nonresorbable monofilament suture. The weight of the sponges inserted was calculated according to the results of the *in vitro* elution tests (see Results) so that the dose of the administered rifampin was 25 mg/kg. Concerning the C group (*N* = 8), 25 mg/kg of rifampin was administered s. c. to the sham surgery pocket in the form of a solution (Eremfat® i.v. RIEMSER Pharma GmbH, An der Wiek 7, 17493 Greifswald–Insel Riems, Germany) in water for the injection application, and the wounds were closed with a nonresorbable monofilament suture. The samples intended for the determination of rifampin levels were drawn 0.5, 1, 2, 4, 6, 10, 22, 34, 46, 58, 70, 82, and 94 hours following the implantation of the sponges. The sample volume was replaced with saline, and the catheters were heparinized following each sample draw.

### 2.9. HPLC Analysis of the Rifampin Levels in the Serum

The determination of rifampin in the PBS solution and the plasma samples was conducted using the Acquity UPLC H-class system (Waters Corporation, Milford, MA). A Kinetex C18 LC column (2.1 mm i.d.×50 mm, 1.3 *μ*m) from Phenomenex (Torrance, California, USA) thermostated at 40°C was used for analysis purposes. The mobile phase consisted of 0.1% of trifluoroacetic acid (solvent A) and acetonitrile (solvent B). The flow rate of the mobile phase was maintained at 0.3 mL/min. The optimized gradient program (min/% B) was 0/20, 1/20, 5/80, 7/80, 8/20, and 11/20. The injection volume was 5 *μ*L, and the samples were maintained at 5°C. Detection was performed by means of a diode array detector (DAD), and the wavelength was set at 340 nm. The samples were deproteinized using acetonitrile prior to the analysis of the plasma samples (the addition of 60 *μ*L of 100% acetonitrile to 20 *μ*L of plasma), and deproteinization was performed in an Eppendorf tube via vortexing for 20 s. The plasma samples were then centrifuged at 16000 × g for 8 min, and 60 *μ*L of the supernatant was transferred to an LC vial. The PBS samples were injected directly into the LC system.

The method was validated in terms of linearity, the limit of detection (LOD), the limit of quantification (LOQ), accuracy, precision, selectivity, recovery, and matrix effects in order to demonstrate that it was suitable for the intended purpose. The selectivity of the method was verified via mass spectrometry operated in the full scan mode (Triple Quad 6460 mass spectrometer; Agilent Technologies, Waldbronn, Germany). Selectivity was monitored via the injection of five plasma samples and one PBS sample. The resulting chromatograms evinced no interfering compound (no *m/z* was observed except the *m/z* corresponding to the rifampin) within the rifampin retention time window. Moreover, the DAD peak purity test for all the analyzed samples was successfully met for the rifampin peak, which ensured high selectivity. Selectivity was thus confirmed independently via both DAD and mass spectrometry. Calibration curves were constructed in both the blank plasma and the PBS solution with seven concentrations by means of the plotting of the peak rifampin area against its concentration. Standard plots were constructed, and the linearity was evaluated statistically by means of linear regression analysis employing the least-squares regression method. The method was linear (coefficients of determination (*R*^2^) of higher than 0.9996) in the concentration range 0.02-100 mg/L for the plasma samples and in the range 0.008-100 mg/L for the PBS samples. The LOD value was 0.004 mg/L for the plasma and 0.001 mg/L for the PBS, determined as the lowest concentration that provided a visible peak in the chromatogram. The LOQ values were the lowest calibration points (0.02 mg/L). The accuracy and precision of the method were evaluated via the measurement of 5 replicates at four different concentrations (0.1; 1; 5; and 50 mg/L) prepared by means of spiking rifampin into the blank plasma and the PBS solution. The accuracy (relative error %) was within ±5.1% and the inter- and intraday precisions (RSD %) were within ±3.9%. These samples were also used as the quality control (QC) samples. The recovery from the plasma samples was evaluated via the comparison of the area of the rifampin standard peak of the pre-protein-precipitation spiked plasma sample with that of the corresponding post-protein-precipitation spiked sample at three concentrations (0.05, 1, and 50 mg/L). The recovery ranged from 97.9 to 100.3%. The matrix effect was evaluated at two concentration levels (0.05 and 50 mg/L) for six plasma samples and one PBS sample. Concerning the plasma samples, it was determined by comparing the area of the rifampin standard peak of the post-protein-precipitation spiked plasma sample with that of the 80% acetonitrile (without the matrix effect), while with respect to the PBS samples, it was determined by comparing the area of the rifampin standard peak that spiked into the PBS sample with that which spiked into pure water. The matrix effect ranged from 97 to 101%. This result proved that neither the plasma matrix nor the PBS exerted a significant effect on the reliable quantification of the rifampin. In order to assess the validity of the analytical method, calibration was performed every day prior to the measurement of the samples, and the quality control samples were injected after each 6^th^ sample.

### 2.10. Histological Examination

Following the euthanasia of the animals, tissue samples (the whole area with the implanted sponge and the surrounding tissue) were surgically removed and submitted for further histological analysis. The tissue samples were macroscopically described and dissected into three and/or four parts (cross-sections). We employed classical histotechnological processing (fixating, dehydrating, tissue clearing etc.) and prepared standard formalin-fixed paraffin embedded blocks. We applied the following three distinct staining techniques: (1) standard hematoxylin and eosin combined with the Weigert's resorcin-fuchsin method for the staining of elastic fibers, (2) Picrosirius red for the identification of collagen fibers combined with the Weigert's resorcin-fuchsin method for the staining of elastic fibers, and (3) Alcian blue (pH 2.5) staining combined with the Periodic Acid Schiff (PAS) reaction.

### 2.11. Statistical Analysis

If either the assumption of normality or homoscedasticity was violated, the Mann-Whitney *U* test for two-sample comparisons was applied. Otherwise, ANOVA and the *t*-test were used with the values expressed as the mean ± standard deviation (SD). The data in the contingency tables was evaluated using the Fisher exact test. GraphPad Prism (Graph software, La Jolla, CA, USA) was used for the visualization of the microbiological, rifampin release, and *in vivo* tests. The thickness of the sponges and the tangent elastic moduli for the selected deformation states were compared with the inequality of the sample variance via the two-sample *t*-test (results presented as arrowheads). Statistical significance was accepted at *p* ≤ 0.05.

## 3. Results

### 3.1. Scanning Electron Microscopy

**Figure 5:**
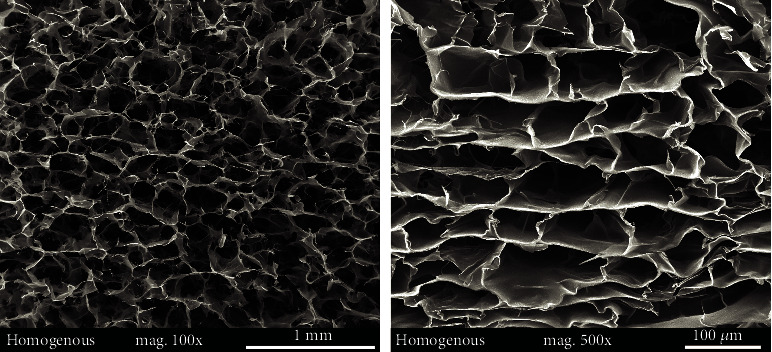
Representative SEM images of the homogeneous collagen sponges.

**Figure 6:**
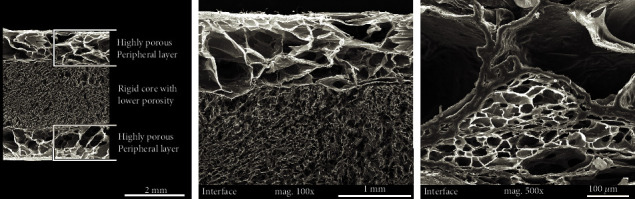
Scheme and representative SEM images of the sandwich sponges with details of the interface between the rigid core with lower porosity and the highly porous peripheral layers.

Representative SEM images of the homogeneous and sandwich sponges are presented in Figures 5 and 6.  Figure 6 shows SEM images of the interface between the highly porous peripheral layer and the rigid core with lower porosity. The interface between the layers with different degrees of porosity does not evince any signs of delamination.

### 3.2. Mechanical Properties of the Collagen Sponges

The thicknesses of the studied samples are depicted in Figure 4. The results demonstrated that during the incorporation of the rifampin into the H and S-F collagen sponges which, in the lyophilized state evinced a lower collagen concentration, the thickness of the samples decreased. No changes in thickness were observed, however, with respect to the sandwich design (S) and sandwich core (S-C) sponges.

**Figure 7:**
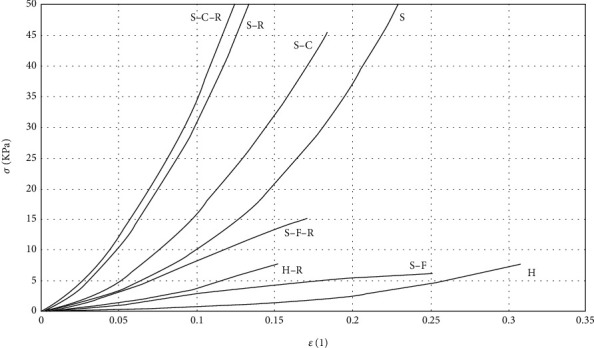
Typical stress-strain curves obtained in uniaxial tensile test.

**Figure 8:**
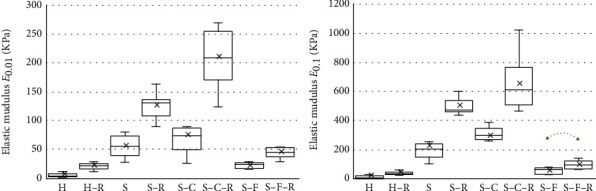
Tangent elastic moduli *E*_0.01_ and *E*_0.1_ obtained for all the groups (*N* = 10). The arrows denote pairs of identical sponges with or without rifampin and without statistically significant differences (*t*-test, *p* > 0.05).

Typical stress-strain curves obtained in uniaxial tensile test are shown in Figure 7. The tangent moduli *E*_0.01_ and *E*_0.1_ were employed to clarify whether the mechanical properties of collagen sponges depend on the design of the sponge. A comparison of H versus S revealed that the sandwich-structured sponges were significantly stiffer than the homogeneous collagen sponges and that they were able to bear higher loads. The same results were also obtained for the H-R versus the S-R sponges. Moreover, a higher degree of stiffness was also determined for the S-C and S-F material when compared to the homogeneous sponges.  Figure 8 provides specific numerical values.

With regard to the effect of rifampin, the comparisons based on both the elastic moduli revealed that the samples with rifampin were stiffer than those with no added antibiotics. The results attained statistical significance (*p* < 0.05) in all cases with the sole exception of S-F versus S-F-R.  Figure 8 displays the results obtained in the form of box plots.

### 3.3. Rifampin Stability Tests

**Figure 9:**
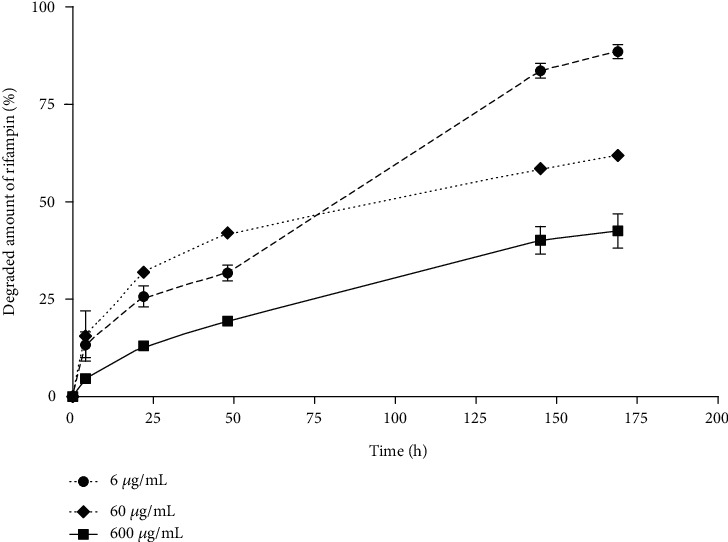
Mean ± SD of the percentage of rifampin that degraded over time when dissolved in PBS and incubated at 37°C protected from light.

Figure 9 illustrates the stability of rifampin in PBS at 37°C protected from light. The rifampin degradation process in the 600 *μ*g/mL and 60 *μ*g/mL concentrations slowed down and became linear following a rapid decline in concentration within the first 2 days. This may have been due to the fact that equilibrium between the rifampin and its degradation products was attained after this time period. The degradation of the rifampin in the 6 mg/mL concentration proceeded markedly more rapidly. After 7 days, approximately 40%, 60%, and 90% of the rifampin degraded in the 600 *μ*g/mL, 60 *μ*g/mL, and 6 *μ*g/mL solutions, respectively.

### 3.4. Rifampin Release Test

**Figure 10:**
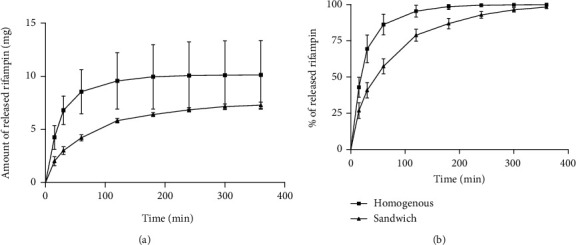
In vitro rifampin release tests: (a) mean ± SD of the absolute amount of released rifampin over time; (b) mean ± SD of the percentage of released rifampin over time (100% = amount eluted at 24 hours).

Figure 10 illustrates the kinetics of the release of rifampin from the sponges. It was determined that the amount of the released rifampin was more variable in the H sponges than in the S sponges (Figure 10(a)). The variability originated from the significant variability in the weight of the samples. Although the samples were in the same size (10 × 20 mm), they had different thicknesses and, therefore, different weights as revealed by the mechanical testing results (Figure 4). A linear correlation was determined between the weights and the amount of rifampin released from the H samples (data not shown). The variability of the amount of rifampin in the S sponges was lower; indeed, the H sponges had generally higher contents of rifampin than the S sponges. It was determined that the amount of rifampin was 30.5 ± 4.6% and 17.3 ± 2.4% for the H sponges and S sponges, respectively. This represented a lower amount of rifampin than was expected since the weight ratio of the rifampin added during the preparation of the sponges was 4 : 5 (rifampin : collagen). The difference was possibly due to the loss of part of the rifampin during the lyophilization process.

Since the amount of rifampin released from the sponges during the final medium exchange after 24 hours of incubation was below the quantification limit (0.05 mg/L) for most of the samples, the total amount of rifampin released after 24 hours of incubation and nine PBS exchanges was accepted as the total amount of rifampin in the sponges.  Figure 10(b) provides a comparison of the rifampin release kinetics of the H and S sponges. The release of rifampin from the S sponges was slightly slower than that from the H sponges.

### 3.5. Disc Diffusion Tests

**Figure 11:**
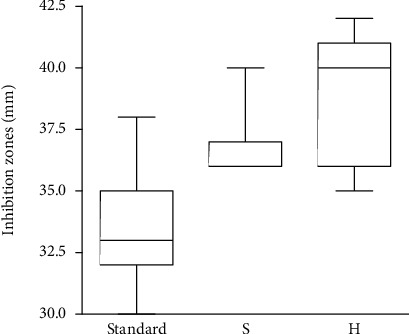
Disc diffusion tests. Comparison of the inhibition zones of the standard microbiological discs with the sandwich-structured sponges (S) and the homogeneous collagen sponges (H).

Figure 11 illustrates the results of the disc diffusion tests with standard microbiological discs with rifampin and the tested sponges (S and H).

The disc diffusion zones proved that the sponges contained rifampin in its active form and that they released the rifampin in amounts high enough to cause the growth inhibition of the MRSA strain. Both the tested groups exhibited significantly greater inhibition zones than the standard (*p* values of lower than 0.001 for both sponges compared to the standard). The difference in the diameter of the inhibition zones between the sponges was not statistically significant. The variability of the diameters of the inhibition zones was higher for the H sponges than for the S sponges.

### 3.6. In Vivo Pharmacokinetic Tests

**Figure 12:**
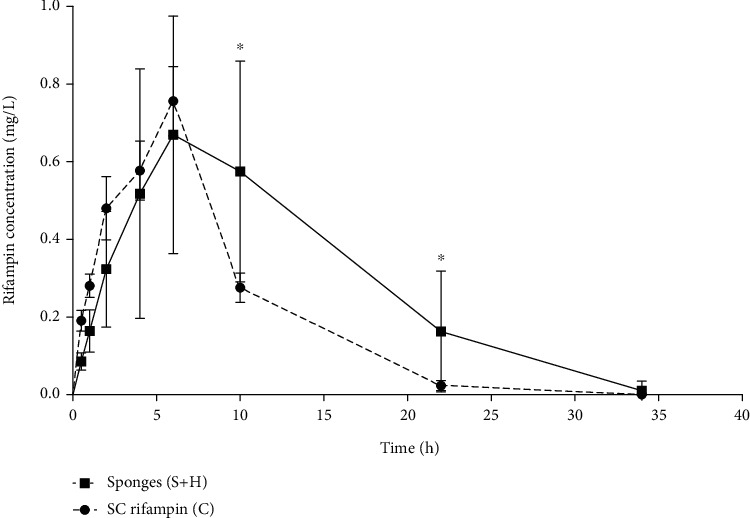
Mean ± SD rifampin plasma levels in rats with rifampin-containing sponge implantations (S+H group) and with the s. c. administration of the rifampin solution (C group). The dose was 25 mg/kg for both groups. ^∗^ Mann-Whitney test, *p* < 0.05.

As the sponges that were administered s. c. to rats were cut to contain the same rifampin amount and values of rifampin plasmatic levels were not statistically significantly different in any of the measured time points for S and H groups, we analyzed both sponges as one group (S+H, *N* = 12) and compared it with control group with SC rifampin (C, *N* = 8).  Figure 12 shows the comparison of plasmatic levels of rifampin in S+H and C groups.  Table 1 shows the main pharmacokinetic parameters in S+H and C groups.

A significant difference was observed between the groups with respect to the AUC. The C group had a lower degree of exposure (AUC) to the rifampin, which may have been caused by interindividual variability. The mean *T*_max_ occurred significantly later in the S+H group than it did in the C group, which proved a certain delay in terms of absorption in the S+H group (Table 1).  Figure 12 depicts the comparison of processes of S+H and C groups' mean plasmatic concentration curves. It is clearly shown that sponges slow down the rifampin release, and most of the sponges sustained measurable and clinically meaningful concentration of rifampin for 24 hours. Nevertheless, the variability of rifampin concentrations in most of the time points is higher in the case of sponges than in the case of s. c. administration.

### 3.7. Histological Examination

**Figure 13:**
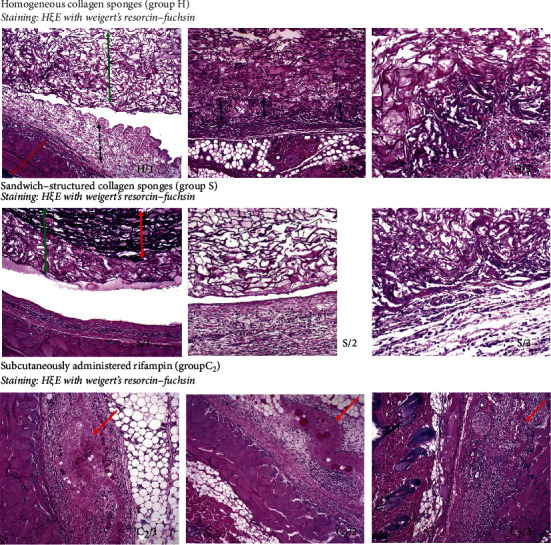
Histological examination of tissues surrounding the area of sponges (groups H and S) or s. c. (group C2) rifampin administration.

**Figure 14:**
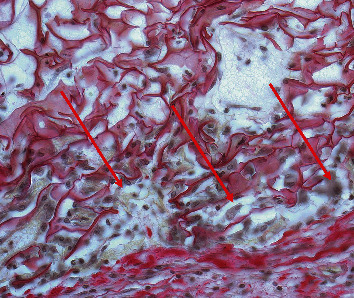
Sample from animal in group S (sandwich-structured collagen sponges). Detail of cells (arrows) penetrating between the structures of the scaffold in the adhesion zone of the sponge and the animal connective tissue (staining: Picrosirius red with Weigert's resorcin-fuchsin).

The histological examination demonstrated the excellent biocompatibility of the sponges with the surrounding tissue (Figures 13 and 14). In Figure 13 (H/1), the upper part (green arrow) was formed by the H sponge material that was implanted within the subcutaneous tissue. The dark blue arrow highlights a mild connective tissue reaction on the material that forms a normal part of the healing process represented by edematous granulation tissue around new thin-walled capillaries and the formation of small prominences and with no inflammation. Muscles can be observed (red arrow) in the bottom left corner of the image. Only one animal in the H group exhibited significant inflammation and hemorrhage within the granulation tissue; otherwise, normal healing process occurred and the cells readily entered the sponge scaffold and grew within the sponges. The majority of the animals in group H evinced segments with the adhesion of the sponge material and the rat connective tissue as is depicted in Figure 13 (H/2). Cells can be observed penetrating from the connective tissue (black arrows) into the sponge structure. Figure 13 (H/3) depicts detail of cells penetrating into the sponge scaffold.

Figure 13 (S/1) depicts a reaction to the sandwich-structured sponge in a similar way to that shown in H/1. The upper part (green arrow) was formed by the sponge material with a different central component (red arrow) implanted within the subcutaneous tissue with a very mild tissue reaction. Figure 13 (S/2) shows detail of edematous granulation tissue with scattered inflammatory cells (normal tissue reaction) at the bottom of the image. In Figure 13 (S/3), the scaffold is seen in the upper part of the image and a layer of scaffold structures can be observed in the center with cells in the adhesion zone of the sponge material and the connective tissue. Edematous granulation tissue with scattered inflammatory cells can be seen at the bottom of the image. The detailed image of cells penetrating into the implanted sandwich-structured sponge is depicted in Figure 14.

A much more remarkable inflammatory reaction to the rifampin solution administered s. c. was observed (group C, data not shown), and since this result may have been caused by administration via surgery, we decided to perform additional tests further on 6 female Wistar rats (group C2); the results of which revealed that the rifampin solution was tolerated well without the occurrence of any remarkable local reactions. Figure 13 (C2/1) depicts a mild interstitial reaction in the center of the picture with a small area of necrotic tissue (red arrow) surrounded by a thin layer of granulation connective tissue with scattered inflammatory cells. Mild inflammation can also be observed in the fat tissue adjacent to the area around the injection site. In Figure 13 (C2/2), injection site (red arrow) with the formation of minor necrosis surrounded by mildly inflamed reactive granulation connective tissue is depicted. This represents the normal healing process. Figure 13 (C2/3) depicts the injection site in the center of the upper part of the image. The mild connective tissue reaction represents a normal part of the healing process with the formation of granulation tissue accompanied by mild inflammation.

## 4. Discussion

The study presented two types of collagen sponges with the addition of rifampin with the potential to serve as wound dressings that prevent postoperative wound infection. The first type consists of a homogeneous collagen sponge and the second a sandwich-like sponge comprising a dense core layer surrounded by porous layers that both enhance adhesion to the surrounding tissues and provide for effective fluid absorption from the wound.

The results of the uniaxial tensile tests conducted on the homogeneous collagen sponges, the sandwich-structured sponges, and individual components of sandwich-structured samples (core and face sheet) suggested that from the mechanical point of view, the design of the sponge constitutes an important factor. The experiments on hydrated sponges clearly demonstrated that the sandwich structure is significantly stiffer than the homogeneous material and, therefore, potentially provides for the enhanced protection of healing wounds. The same results were determined following the study of the effect of rifampin. In particular, following the application of rifampin, the sandwich design was observed to bear a higher load than the homogeneous sponges. Thus, it was concluded that from the handling point of view, the sandwich-structured collagen sponges appear to provide better candidates for further research and that the addition of rifampin significantly alters the mechanical properties of collagen sponges (Figure 8).

Moreover, the structure of the sandwich sponges with respect to thickness was found to be more uniform than that of the homogeneous sponges (Figure 9), and even though the sandwich sponges contained lower amounts of rifampin in the same unit area than did the homogeneous sponges, the amount of rifampin was significantly less variable (Figure 10). With the exception of differences in terms of rifampin content variability, the two sponges evinced similar rifampin release properties (Figure 10). We proved that all the rifampin was completely released from the sponges after 6 hours of *in vitro* eluting testing. This is in contradiction to the results obtained by Parker et al. [14] who soaked their collagen sponges in a rifampin solution and determined that 25% of the rifampin was not released during release testing. Even though the authors suggested that the rifampin bonded within the sponges, the most probable explanation for this observation is that the rifampin degraded during testing in line with our observation that 10-30% of the rifampin degraded during the first day at 37°C (Figure 9).

When compared *in vivo*, the release of rifampin from the sponges was slower than that from the direct s. c. application of the rifampin solution (Figure 12), and moreover, the sponges maintained systemic rifampin levels at above 0.2 mg/mL for at least 22 hours, which is a higher level than the MIC for streptococci and staphylococci according to the European Committee on Antimicrobial Susceptibility Testing database (https://mic.eucast.org, cited in 01/2020). This was not attainable via the administration of a single dose of rifampin s. c. Rifampin concentrations are much higher in the location of absorption than in the systemic circulation, and rifampin has been proved to be effective in terms of postsurgical wound infection prevention when administered locally [5]. The sponges presented in this study have the potential to serve as ideal postoperative dressings following surgery in infection-susceptible areas including those colonized by methicillin-resistant *staphylococcus aureus* (MRSA). Moreover, since rifampin readily penetrates biofilms [8–10], we suggest that the presented sponges will be effective when applied locally for the treatment of infected postoperative wounds complicated by the formation of biofilms, especially in cases where the wound requires a new dressing every 24 hours.

We also proved the excellent biocompatibility parameters of the sponges, which is in agreement with previous studies that described carbodiimide-cross-linked collagen sponges derived from the *cyprinidae* family as being highly biocompatible [16]. We also proved in previous research that the immunogenicity of *cyprinus carpio*-coated vascular grafts is comparable to that of collagen of bovine origin [27]. Although some authors have proved rifampin to be cytotoxic in very high concentrations of over 1 mg/mL [28], we proved that rifampin (although classified as a local irritant) does not diminish the excellent biocompatibility of *cyprinus carpio* collagen cross-linked with carbodiimide. The histological studies showed that the edges of both types of sponges were settled by cells that migrated from the surrounding tissues and that the healing process was initiated as soon as after just 5 days (Figures 13 and 14). This is also in agreement with previously conducted studies involving bone healing rat models in which neither rifampin nor vancomycin powder added to a bone fracture proved any signs of healing impairment [29].

One drawback of the sponges presented herein is that they release the antibiotic for 24 hours only. However, it would be extremely complicated to prepare sponges that prolong the release of rifampin beyond 24 hours due to the rapid degradation of rifampin under body conditions (Figure 9).

## 5. Conclusions

The study presented a novel three-layered cross-linked fresh water fish collagen wound dressing *in vivo* that released rifampin for at least 22 hours and which has the potential to serve as an ideal surgical wound infection prevention or treatment approach. Its mechanical properties and the homogeneity of the rifampin content were compared with those of a simple homogeneous collagen sponge and proved to be superior in terms of both these aspects. The dressing demonstrated excellent bioavailability properties and no inhibition of the wound healing process in an animal model.

## Figures and Tables

**Table 1:** Mean ± SD of the rifampin pharmacokinetic parameters in the rats—comparison of the s. c. injection of the rifampin solution in water (C) and the s. c. administered rifampin-containing sponges (S+H).

	*T*_1/2_ [h]	*T*_max_ [h]	*C*_max_ [mg/L]	AUC_0−inf_ [mg∗h/L]
SC sponges (S+H)	6.5 ± 1.5	6.9 ± 0.8	0.8 ± 0.1	11.5 ± 1.5
SC rifampin solution (C)	3.8 ± 0.4	4.5 ± 0.5	0.9 ± 0.1	6.9 ± 0.7
*p* value	0.11	0.03	0.55	0.02

## Data Availability

All data are included in the manuscript. Further details can be obtained upon request to the corresponding author.
